# SASP-mediated cellular senescence following myocardial infarction: from spatiotemporal immune regulation to therapeutic strategies

**DOI:** 10.3389/fimmu.2026.1850084

**Published:** 2026-06-16

**Authors:** Fengmei Zhang, Yuanpeng Liao, Peng Yang, Jiawei Guo

**Affiliations:** 1 Department of Cardiology, The First Affiliated Hospital of Yangtze University, Jingzhou, China; 2 Department of Pharmacology, School of Medicine, Yangtze University, Jingzhou, China

**Keywords:** cellular senescence, immune regulation, inflammation, myocardial infarction, senescence-associated secretory phenotype

## Abstract

Currently, the primary causes of death following myocardial infarction include sudden cardiac death, malignant arrhythmias, and acute heart failure, all resulting from myocardial necrosis caused by coronary artery occlusion. Due to population aging and lifestyle changes, the global number of patients with myocardial infarction is expected to continue to rise. Cellular senescence refers to the permanent arrest of cell proliferation in response to stress stimuli; it serves as a crucial tumor defense mechanism and is closely associated with tissue aging and chronic inflammation. The senescence-associated secretory phenotype (SASP) is one of the most characteristic features of senescent cells. Cardiac cells develop a SASP and secrete SASP factors in response to stimuli such as oxidative stress, DNA damage, and hypoxia, playing a key role in immune regulation and tissue repair following myocardial infarction. The SASP exhibits marked spatiotemporal heterogeneity following myocardial infarction: during the acute phase, it contributes to inflammatory amplification and immune cell recruitment; during the subacute phase, it is involved in inflammation resolution, matrix remodeling, and scar formation; and during the chronic phase, it promotes chronic inflammation, paracrine senescence, pathological fibrosis, and cardiac dysfunction. Spatially, the SASP influences scar stabilization in the infarct zone, inflammation–electrophysiological coupling in the border zone, and compensatory remodeling in the distal region. The sustained expression of the SASP is a major driver of adverse ventricular remodeling and excessive fibrosis following myocardial infarction. Therefore, targeting senescent cells and persistent, pathological SASP represents a highly promising therapeutic strategy in the field of cardiovascular regenerative medicine. This review will discuss senescence in different cell types following myocardial infarction, the spatiotemporal heterogeneity of the immune response mediated by the SASP after myocardial infarction, and the immune cells regulated by the SASP.

## 1 Introduction

**Figure 1:**
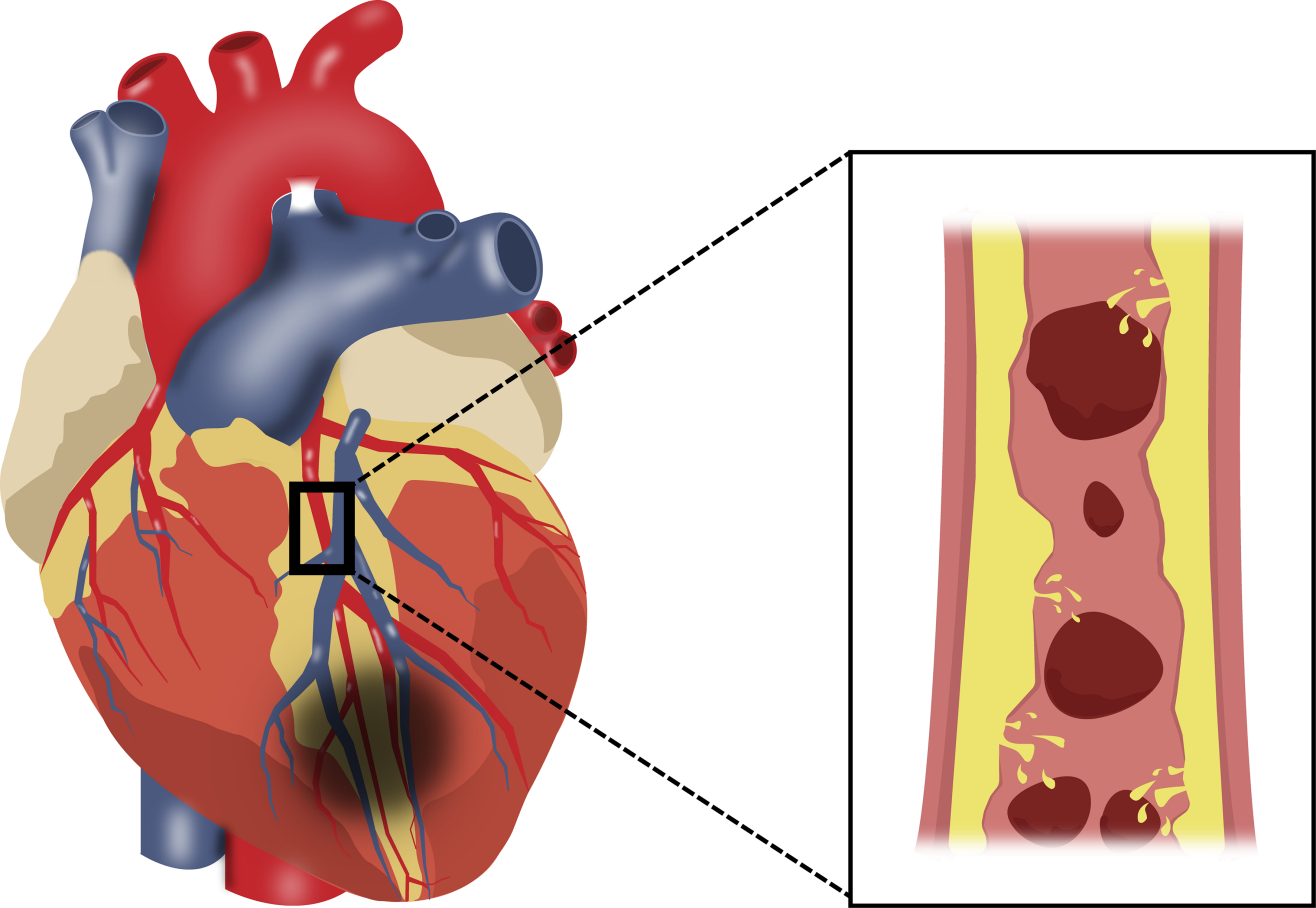
MI. acute occlusion of a coronary artery due to plaque rupture, erosion, or thrombus formation leads to severe ischemic necrosis of the cardiomyocytes in the supplied region. The necrotic area subsequently triggers a vigorous inflammatory response and is replaced by non-contractile fibrous scar tissue under the influence of fibroblasts. This process may ultimately result in ventricular remodeling, arrhythmias, or acute heart failure. MI, myocardial infarction.

According to estimates from the Global Burden of Disease (GBD) Study, cardiovascular disease (CVD) has long been among the leading causes of global disease burden and mortality. In 2023, the global prevalence of CVD reached 626 million cases, nearly doubling from 311 million in 1990; it also accounted for 437 million disability-adjusted life years (DALYs), significantly higher than the 320 million recorded in 1990 (1). From a temporal trend perspective, global CVD DALYs in 2023 were approximately 1.37 times those of 1990, indicating that the overall disease burden continues to rise. Myocardial infarction (MI) is the necrosis of heart muscle tissue caused by acute and sustained ischemia and hypoxia in the coronary arteries—the blood vessels that supply the heart (**Figure 1**) (2). The mechanism of coronary artery occlusion involves various pathological processes, the most common of which is the rupture or erosion of an atherosclerotic plaque, particularly in ST-segment elevation myocardial infarction (STEMI) (3). When the fibrous cap of a plaque ruptures, exposing the underlying lipid core, it rapidly activates platelet aggregation and the coagulation system, leading to thrombus formation that obstructs the coronary artery (4). Additionally, plaque erosion—where the endothelial cells on the plaque surface are damaged but the plaque itself remains intact—can also trigger thrombus formation (5). Other mechanisms include intraplaque hemorrhage (IPH), which causes rapid volume expansion and compression of the lumen; isolated coronary artery spasm (CAS); coronary microvascular dysfunction (CMD); spontaneous coronary artery dissection (SCAD); and embolism from thrombi or foreign bodies originating elsewhere (6–9). Whether cardiomyocytes (CMs) sustain irreversible damage depends critically on the duration of ischemia following coronary occlusion. In the early stages of ischemia, myocardial cells may exhibit functional impairment, primarily manifested as a decline or loss of contractile function; however, no irreversible structural damage has yet occurred. If blood flow is rapidly restored within a short period—a process known as reperfusion—myocardial cells typically retain the potential for recovery. Nevertheless, some myocardial tissue may continue to exhibit temporary, reversible contractile dysfunction even after blood flow is restored; this phenomenon is referred to as myocardial stunning (10). Once the duration of ischemia exceeds 30 minutes, cardiomyocytes begin to sustain irreversible damage. During the irreversible necrosis phase (30 minutes to 6 hours), myocardial cells exhibit a wavefront phenomenon; cell death rapidly evolves from initial “focal” or “limited-area” irreversible damage into extensive necrosis spreading in a wave-like pattern. The necrotic area progresses from the endocardium to full-thickness necrosis, with cellular structures disintegrating to form a transmural myocardial infarction. Subsequently, inflammatory responses and repair processes are initiated, ultimately resulting in replacement by non-contractile scar tissue, leading to ventricular remodeling and reduced cardiac function (11). If reperfusion is achieved promptly within 30 minutes to 3 hours of ischemia [e.g., via percutaneous coronary intervention (PCI)], a significant portion of the myocardium can be salvaged (12). Globally, the incidence of myocardial infarction varies significantly. In high-income countries, the overall incidence is declining, whereas in developing countries, it is rising and showing a trend toward younger age groups (13). Therefore, timely diagnosis and evidence-based treatment are essential to reduce mortality and improve clinical outcomes after myocardial infarction.

Following a myocardial infarction, the heart undergoes a complex process of pathological remodeling. During the acute phase, necrotic cells trigger a strong inflammatory response, and the infarct area expands outward due to mechanical stress (14, 15). In the intermediate phase, fibroblasts are activated and secrete large amounts of collagen, forming hard fibrous scar tissue to fill the necrotic area; however, this simultaneously leads to compensatory hypertrophy of the non-infarcted myocardium, gradual enlargement of the ventricular cavity toward a spherical shape, and reduced pumping efficiency (16, 17). In the chronic phase, the sympathetic nervous system and the renin–angiotensin–aldosterone system remain activated, further exacerbating cardiac workload and fibrosis. Concurrently, electrophysiological remodeling increases the risk of malignant arrhythmias, which may ultimately lead to heart failure (18). The core challenge in cardiac repair following myocardial infarction lies in the fact that adult cardiomyocytes have extremely low proliferative capacity; dead cardiomyocytes cannot regenerate and are replaced by fibrous scar tissue, leading to impaired cardiac mechanical function and electrical conduction, which in turn triggers ventricular arrhythmias and heart failure (19, 20). Furthermore, the precise regulation of inflammatory and immune responses, the restoration of blood supply to the infarcted area, and the safety of clinical translation for any potential repair strategies are all challenges that need to be overcome in current research and treatment.

Cellular senescence refers to the irreversible loss of a cell’s ability to divide and proliferate, resulting in a stable state of growth arrest. Senescent cells accumulate with advancing age and are associated with age-related pathological conditions (21). A hallmark feature of senescent cells is the upregulation of the major cyclin-dependent kinase (CDK) inhibitors p21^CIP1 and p16^INK4A. These proteins block the transcription of E2F target genes by inhibiting the activity of CDK-cyclin complexes and reducing the phosphorylation levels of Rb family proteins, ultimately leading to permanent cell cycle arrest (22). Furthermore, the response to unrepaired DNA damage, through the activation of the p53 pathway, is also one of the key mechanisms inducing cellular senescence (23).

Although senescent cells lose their ability to proliferate, they still perform important biological functions by secreting many bioactive molecules; this secretory characteristic is known as the senescence-associated secretory phenotype (SASP) (24). The composition of the SASP is complex, including various interleukins, chemokines, cytokines, proteases, growth factors, and non-coding RNAs among (25). The SASP plays a dual role in tissue repair and regeneration. In the early stages of acute injury, such as myocardial infarction, the SASP helps limit fibrosis, promote angiogenesis, and prevent excessive scarring, thereby exerting a positive influence on tissue repair (26). However, with the continued accumulation of senescent cells, the SASP may transform into a driver of chronic inflammation, exacerbating tissue damage, promoting fibrosis and functional decline, and even inducing neighboring cells to enter a senescent state, forming the so-called “bystander effect” (27). Concurrently, extracellular vesicles (EVs), as a newly identified insoluble component of the SASP, can transmit senescence signals between cells by carrying molecules such as proteins and nucleic acids and act in concert with soluble SASP factors to amplify inflammatory responses (28, 29). Therefore, the SASP is not only a key marker of cellular senescence but also a critical regulator of age-related pathological processes.

This review will focus on the senescence of different cell types following myocardial infarction, the spatiotemporal heterogeneity of the immune response mediated by the SASP in myocardial infarction, the relationship between the SASP and specific immune cells, and therapeutic strategies.

## 2 Senescence of different cell types after MI

**Figure 2:**
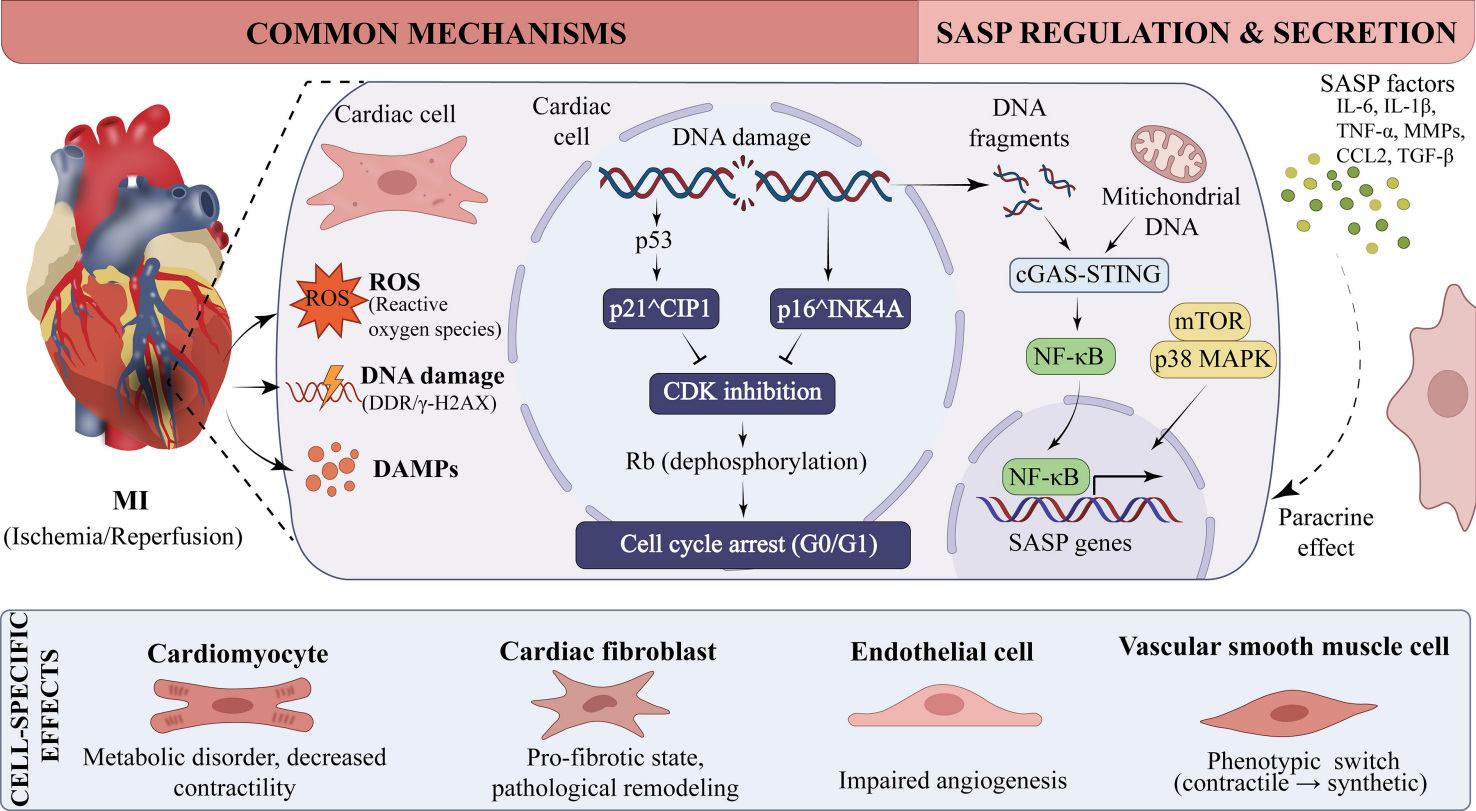
Ischemia–reperfusion injury, ROS accumulation, and DNA damage following MI trigger cell cycle arrest pathways centered on p53/p21^CIP1 and p16^INK4A, inducing various cardiac cells to enter senescence and maintaining the sustained secretion of the SASP through molecular programs such as cGAS–STING, NF-κB, and mTOR. Against this backdrop, different cell types exhibit significant phenotypic heterogeneity: cardiomyocytes display impaired contractile function and mitochondrial homeostasis imbalance, fibroblasts regulate scar formation and matrix remodeling by secreting pro-fibrotic factors and MMPs, ECs exhibit impaired barrier function and reduced angiogenic capacity, and VSMCs lead to decreased vascular compliance through phenotypic transformation. Through SASP-mediated paracrine effects, these cells collectively shape the pathophysiological environment of the post-infarction heart. Abbreviations: MI, myocardial infarction; ROS, reactive oxygen species; DAMPs, damage-associated molecular patterns; Rb, retinoblastoma protein; DDR, DNA damage response; NF-κB, nuclear factor-kappa B; SASP, senescence-associated secretory phenotype; mTOR, mammalian target of rapamycin; MMPs, matrix metalloproteinases; γ-H2AX, phosphorylated histone H2AX.

Cellular senescence constitutes a crucial biological foundation of cardiac aging. As age advances or the duration of inflammation is prolonged, the body’s capacity to clear senescent cells diminishes. In an infarcted heart, ischemia, reperfusion injury, oxidative stress, inflammatory stimuli, mechanical stress, and the extracellular matrix (ECM) collectively induce senescence in various types of cardiac cells (**Figure 2**). Immunosenescence can reduce the body’s efficiency in clearing senescent cells, leading to their continued accumulation in the infarcted heart and providing a cellular basis for subsequent SASP-mediated immune regulation and ventricular remodeling.

### 2.1 Common mechanisms of cellular senescence following myocardial infarction

Although different types of cardiac cells exhibit distinct senescence phenotypes following myocardial infarction, there are common molecular mechanisms underlying the induction and maintenance of cellular senescence. This mechanism can be summarized as two sequential but not entirely equivalent processes: first, ischemia–hypoxia, reperfusion-induced oxidative stress, and DNA damage induce cells to enter a stable senescent or senescence-like state. Subsequently, pathways such as NF-κB, C/EBPβ, GATA4, cGAS–STING, mTOR, and p38 MAPK–MK2 collectively maintain the transcriptional, translational, and secretory activities of the SASP. During a myocardial infarction, coronary artery occlusion causes cardiac tissue to undergo severe ischemia–hypoxia. Subsequently, blood flow is restored through PCI or thrombolytic therapy. Although this can salvage some of the dying myocardium, the sudden influx of oxygen during reperfusion triggers severe oxidative stress (30). This process generates large amounts of reactive oxygen species (ROS), which can damage lipids, proteins, nuclear DNA, and mitochondrial DNA, becoming a major contributing factor to stress-induced premature senescence in various cardiac cells following myocardial infarction (31).

ROS can penetrate the cell nucleus, inducing double-strand breaks in DNA, thereby causing significant DNA damage (32). Following DNA damage, the cellular DNA damage response (DDR) pathway is rapidly activated; markers such as the phosphorylation level of γ-H2AX are significantly elevated in senescent cardiomyocytes, further amplifying cardiac cellular senescence (33). Persistent DNA damage signals potently activate the two core cell cycle arrest pathways: p53/p21^CIP1 and p21^CIP1/Rb (34). As the central hub of the DNA damage response, p53, upon phosphorylation and activation, upregulates the transcription of its downstream target gene, p21^CIP1 (35). As a cyclin-dependent kinase inhibitor, p21^CIP1 binds to and inhibits the activity of cyclin complexes, blocking the phosphorylation of Rb protein and thereby inducing permanent cell cycle arrest in the G0/G1 phase (36). Concurrently, the p16^INK4A pathway is frequently activated, particularly in the context of aging hearts or chronic injury. By inhibiting CDK4/6, p16^INK4A maintains Rb in a hypophosphorylated state, further consolidating the cell cycle arrest (37, 38). Consequently, cardiac cells in the infarct zone and the peri-infarct region, even if they survive, mostly enter this functionally abnormal and non-dividing senescent state.

In addition, the inflammatory response triggered by the massive release of damage-associated molecular patterns (DAMPs) following myocardial infarction creates a microenvironment that induces cardiac cell senescence (39, 40). DAMPs can activate the innate immune response, promoting the infiltration of immune cells such as neutrophils and monocytes/macrophages (41). They also induce the release of pro-inflammatory cytokines such as IL-1β, IL-6, TNF-α, CCL2, and TGF-β (42). These inflammatory mediators not only directly exacerbate cardiac cell damage but also induce neighboring cells to enter a senescent state via paracrine mechanisms, forming a mutually reinforcing positive feedback loop (43, 44). Concurrently, multiple factors following myocardial infarction—including calcium homeostasis disruption, mitochondrial dysfunction, changes in mechanical stress, and impaired autophagy—can also drive cardiac cells into an irreversible state of cell cycle arrest, known as senescence (33, 45, 46). Senescent cells typically exhibit elevated senescence-associated β-galactosidase (SA-β-Gal) activity; upregulation of p16^INK4A, p21^CIP1, and p53; accumulation of DNA damage markers such as γ-H2AX; chromatin remodeling; and secretion of the SASP (47). These shared mechanisms provide a molecular basis for understanding how different types of cardiomyocytes enter a senescent state following myocardial infarction.

The SASP is not merely a transient elevation of inflammatory factors but rather a relatively persistent secretory phenotype in senescent or senescent-like cells driven by specific molecular programs. At the molecular level, the initiation and maintenance of the SASP are precisely regulated by a series of signaling pathways. The DDR serves as an upstream trigger for the SASP. Concurrently, GATA4 acts as a bridge between DNA damage and inflammatory signaling, participating in the activation of the NF-κB pathway to amplify the effects of the SASP (48). The cGAS–STING pathway has garnered significant attention in recent years. When mitochondrial DNA leaks or nuclear DNA fragments enter the cytoplasm, they activate cGAS to generate cGAMP, which in turn activates STING, inducing IRF3- and NF-κB-mediated expression of type I interferons and inflammatory factors. This pathway plays a critical role in the recruitment of immune cells following myocardial infarction (49). In myocardial infarction, IL-1 activates the NF-κB pathway, driving the transcriptional expression of pro-inflammatory factors such as IL-6, MCP-1, and matrix metalloproteinase (MMP)-3/8, as well as genes related to matrix remodeling (50). The activation of the mTOR signaling pathway, particularly mTORC1, promotes IL-1α synthesis at the translational level, thereby enhancing the SASP effect; the use of mTOR inhibitors such as rapamycin can effectively alleviate SASP-related inflammation (51, 52). The p38 MAPK–MK2 pathway participates in the regulation of SASP mRNA stability; inhibiting this pathway reduces the expression of factors such as IL-6 and CXCL1 (53, 54). Mitochondrial dysfunction and ROS production not only directly activate NF-κB but also enhance the SASP effect through metabolic reprogramming, further exacerbating the inflammatory response (55).

### 2.2 Cardiomyocytes

Cardiomyocytes constitute most of the cardiac tissue (approximately 80% by volume); therefore, changes in their functional state are sufficient to affect the heart’s overall function. The senescent-like changes that occur after myocardial infarction may serve as an important source of local SASP signaling. Myocardial cell senescence following myocardial infarction is a form of premature senescence induced by severe stress (56). Unlike proliferative cells, most adult cardiomyocytes are in a state of terminal differentiation; therefore, their senescence does not simply manifest as replicative senescence in the traditional sense but rather resembles a stress-induced senescent-like state characterized by functional decline and driven by a combination of persistent DNA damage, telomere damage, mitochondrial homeostasis imbalance, protein homeostasis disruption, and SASP-like secretion (57).

Under the influence of ischemia or reperfusion, oxidative stress, and DNA damage, cardiomyocytes activate the DDR pathway, promoting the senescent cell phenotype mediated by p53/p21^CIP1 and p21^CIP1/Rb (58). Concurrently, telomere-associated DNA damage can also induce cardiac myocyte senescence; even when telomere length has not yet significantly shortened, the accumulation of telomere damage can sustain the activation of DNA damage signaling (59). Senescent cardiomyocytes often exhibit mitochondrial stress, reduced oxidative phosphorylation efficiency, abnormal calcium handling, and upregulation of aging-related genes (60). Concurrently, the contractile and diastolic functions of senescent cardiomyocytes are impaired, suggesting that their senescent phenotype has clear functional implications (61). These characteristics indicate that senescence manifests more as persistent stress and a remodeling of cellular functional states, rather than merely cell cycle arrest.

From a spatial distribution perspective, cardiomyocytes in the infarct core rapidly decrease due to ischemic necrosis or apoptosis, whereas in the infarct border zone and adjacent non-infarct areas, surviving cardiomyocytes are more prone to stress-induced remodeling and may exhibit senescence-like alterations such as p16^INK4A, p21^CIP1, and DNA damage foci (62, 63). Therefore, cardiomyocyte senescence primarily occurs in sublethal injury zones rather than in purely necrotic regions. These senescent cardiomyocytes can release paracrine signals such as IL-6, CCL2, TGF-β, GDF15, and exosomal miRNAs, thereby influencing the state of adjacent fibroblasts, endothelial cells (ECs), and immune cells (64).

### 2.3 Fibroblasts

During cardiac repair following myocardial infarction, senescent fibroblasts play a dual role: they help limit excessive fibrosis while potentially impairing tissue repair capacity, making them a critical regulatory node influencing post-infarction cardiac remodeling. Under normal conditions, fibroblasts are responsible for maintaining the homeostasis of the extracellular matrix. However, following myocardial infarction, stimulated by multiple factors such as ischemia–hypoxia, oxidative stress, the inflammatory microenvironment, and changes in mechanical stress, fibroblasts enter an irreversible state of cell cycle arrest known as senescence (65, 66). Unlike terminally differentiated cardiomyocytes, fibroblasts possess a strong capacity for proliferation and phenotypic conversion; therefore, their senescence more closely resembles stress-induced proliferative arrest in the classical sense (67). This process is precisely regulated by a series of molecular pathways and exhibits highly ordered dynamic characteristics in both time and space.

Aging fibroblasts exhibit increased secretion of matrix-regulating SASP molecules such as TGF-β, Connective Tissue Growth Factor (CTGF), platelet-derived growth factors (PDGFs), MMPs, tissue inhibitors of metalloproteinases (TIMPs), IL-6, and CCL2 (27, 68). In terms of spatial distribution, senescent fibroblasts are primarily concentrated in the infarct zone, while their levels are relatively low in distant non-infarct areas (69). Approximately 3 to 7 days after myocardial infarction, fibroblasts exhibit high proliferation, reaching a peak of activity around day 7, after which they may persist or be cleared under specific conditions (70). Transient, controlled fibroblast senescence may limit excessive myofibroblast proliferation and help prevent excessive scar formation; however, in the presence of persistent inflammation and pro-fibrotic signals, senescence may shift from a reparative regulation to pathological amplification, and persistent senescent fibroblasts may maintain inflammatory and matrix remodeling signals through long-term SASP secretion, promoting pathological fibrosis (26, 71). This spatiotemporal specificity suggests that senescent fibroblasts may play different roles at different stages of cardiac repair, contributing to the stabilization of the infarct zone in the early phase and potentially promoting adverse remodeling in the late phase.

Furthermore, fibroblast senescence following myocardial infarction may also be influenced by NRG1, TGF-β/Smad signaling, mechanosensitive pathways, and macrophage-derived paracrine mediators (69, 72, 73). Furthermore, the CCN1 protein can induce transient fibroblast senescence in damaged myocardium, while inflammatory and pro-fibrotic signals may exacerbate senescence under conditions of sustained stress (74, 75). As fibroblasts are central regulators of ECM synthesis and degradation, their senescent state and changes in the SASP phenotype serve as critical junctures linking the post-myocardial infarction immune response, scar maturation, and ventricular remodeling.

### 2.4 Vascular smooth muscle cells and endothelial cells

Following myocardial infarction, the senescence of vascular smooth muscle cells (VSMCs) and ECs is also a key pathological process driving adverse cardiac remodeling and dysfunction. Located at the interface between blood and myocardial tissue, ECs are among the first non-immune cells to be affected by DAMPs, ROS, inflammatory factors, and altered shear stress following myocardial infarction. Given that ECs are responsible for maintaining the vascular barrier, regulating vascular tone, controlling leukocyte adhesion, and participating in angiogenesis, their senescent phenotype is closely associated with microvascular repair after myocardial infarction (76). In contrast, VSMC senescence is more prominently characterized by alterations in vascular wall structure and phenotypic plasticity rather than mere barrier dysfunction.

A core feature of senescent VSMCs is a phenotypic shift from a contractile to a synthetic phenotype, accompanied by downregulation of contractile markers (α-SMA and SM22α) and upregulation of factors such as osteopontin (OPN) and Runx2 (77, 78). Following myocardial infarction, this VSMC senescence and phenotypic transition may exacerbate coronary and microvascular wall remodeling, reduce vascular compliance, alter local hemodynamics, and adversely affect the restoration of perfusion in the infarct margin (79, 80). In contrast, senescent ECs exhibit reduced proliferation and migration capacity, impaired angiogenesis, increased permeability, decreased nitric oxide (NO) production, reduced eNOS activity, increased oxidative stress, and upregulation of adhesion molecules (81–85). These characteristics suggest that vascular cell senescence may affect microvascular integrity, leukocyte adhesion, and the angiogenic response following myocardial infarction.

The SASP in senescent ECs is more oriented toward inflammation, adhesion, and angiogenesis regulation. The chemokines, adhesion molecule-related signals, and angiogenesis regulators that they secrete can influence leukocyte transendothelial migration and the efficiency of microvascular repair (86). Senescent VSMCs, in turn, may contribute to vascular wall remodeling, reduced compliance, and altered microcirculatory stability by releasing MMPs, inflammatory factors, and calcification-related molecules (87).

Although both VSMCs and ECs may contribute to the development of the SASP in the infarcted heart, their functional consequences vary depending on anatomical location and disease stage.

## 3 SASP-mediated immune regulation: spatiotemporal heterogeneity

Studies have indicated that SASP-mediated immune regulation following myocardial infarction is by no means a homogeneous biological response but rather exhibits highly complex spatiotemporal heterogeneity.

### 3.1 The conceptual boundaries of SASP and its distinction from the acute inflammatory response

The inflammatory response following myocardial infarction exhibits distinct phases and multiple sources. Although inflammatory factors such as IL-6, TNF-α, IL-1β, CCL2, and CXCL1/2 are often listed as components of the SASP, during the acute phase of myocardial infarction, the primary source of these factors is not necessarily senescent cells. Following acute ischemic necrosis, massive myocardial cell death and the release of DAMPs can rapidly activate neutrophils, monocytes/macrophages, ECs, and fibroblasts, inducing a burst of inflammatory cytokine release (88). Therefore, elevated levels of IL-6, TNF-α, or IL-1β during the acute phase do not, by themselves, prove that these factors originate from senescent cells or constitute the SASP in a functional sense.

From a pathobiological perspective, the core significance of the SASP does not lie in the transient peak of inflammatory factors during the acute phase but rather in the persistent presence of senescent cells during the subacute and chronic phases, which leads to the long-term, low-level, paracrine release of inflammatory factors, chemokines, growth factors, proteases, and extracellular vesicles. This process sustains chronic inflammation, induces senescence in neighboring cells, interferes with immune clearance, and drives ventricular remodeling. Therefore, in studies of myocardial infarction, a distinction should be made between “acute injury-related inflammatory cytokine release” and “persistent SASP originating from senescent cells”. The former is primarily driven by DAMPs, ischemia–reperfusion injury, and innate immune activation, constituting an early response essential for initiating tissue clearance and repair. The latter manifests more as a chronic secretory phenotype resulting from the failure to promptly clear senescent cells, serving as a key mechanism promoting adverse remodeling and the progression of heart failure.

Consequently, the relative contributions and functional shifts of the SASP across different post-MI stages may manifest the following: in the acute phase, the SASP may act as one of multiple inflammatory sources involved in stress amplification and immune cell recruitment; in the subacute phase, the SASP participates in inflammation resolution, matrix remodeling, and scar formation; and in the chronic phase, persistent SASP becomes a major driver of chronic low-grade inflammation, paracrine senescence spread, fibrosis, and worsening cardiac function.

### 3.2 Temporal heterogeneity

**Figure 3:**
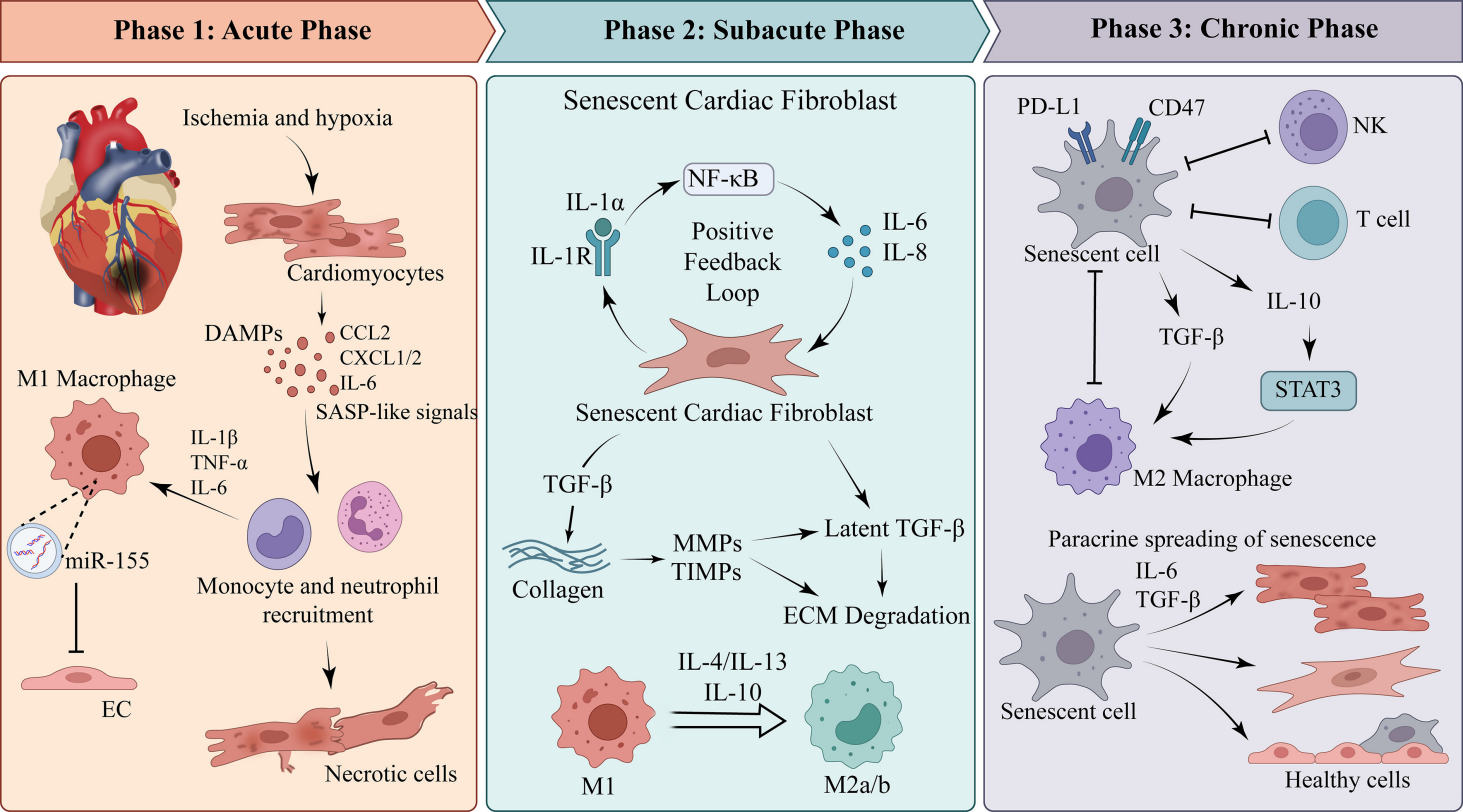
SASP plays a dynamic regulatory role at different stages following myocardial infarction. During the acute phase, it primarily contributes to inflammation amplification and immune cell recruitment; during the subacute phase, it is involved in inflammation resolution, matrix remodeling, and scar formation; and during the chronic phase, persistent SASP drives low-grade inflammation, the paracrine spread of senescence, fibrosis, and worsening cardiac function. Abbreviations: MI, myocardial infarction; SASP, senescence-associated secretory phenotype; DAMPs, damage-associated molecular patterns; CCL2, chemokine ligand 2; CXCL1/2, chemokine ligand 1/2; EC, endothelial cell; NF-κB, nuclear factor-kappa B; TIMPs, tissue inhibitors of metalloproteinases; MMPs, matrix metalloproteinases; ECM, extracellular matrix; PD-L1, programmed death-ligand 1; CD47, cluster of differentiation 47; NK, natural killer cells; IL, interleukin; TNF-α, tumor necrosis factor-alpha; TGF-β, transforming growth factor-beta.

Cardiac repair and remodeling following myocardial infarction is a highly dynamic pathophysiological process in which the SASP plays a crucial role. Recent studies have shown that the SASP exhibits significant heterogeneity at different time points after myocardial infarction. The SASP exhibits stage-dependent functional shifts following myocardial infarction: during the acute phase, it primarily contributes to inflammation amplification and immune cell recruitment; during the subacute phase, it contributes to inflammation resolution, matrix remodeling, and scar formation; and during the chronic phase, persistent SASP drives low-grade inflammation, the spread of paracrine senescence, fibrosis, and worsening cardiac function (**Figure 3**). The core mechanisms underlying this functional transition lie in the persistent presence of senescent cells, imbalances in immune polarization, and the spread of paracrine senescence.

#### 3.2.1 Acute phase of MI

During the acute phase of myocardial infarction—typically defined as the hours to days following onset—myocardial ischemia leads to extensive myocardial cell necrosis, resulting in the release of DAMPs that rapidly activate the local innate immune response (89). The primary drivers of the inflammatory response during this phase are not mature, persistent SASP but rather DAMPs, ischemia–reperfusion injury, ROS production, complement activation, disruption of the local endothelial barrier, and rapid activation of the innate immune system (90). These injury signals rapidly induce the recruitment of neutrophils and inflammatory monocytes to the infarct area and promote the release of inflammatory mediators such as IL-1β, IL-6, TNF-α, CCL2, and CXCL1/2 by macrophages, endothelial cells, fibroblasts, and surviving cardiomyocytes (88). Therefore, the elevation of these pro-inflammatory factors during the acute phase cannot be simply equated with the SASP originating from senescent cells, nor can it serve as direct evidence that the SASP dominates the acute inflammatory response.

Nevertheless, acute ischemia–hypoxia, reperfusion-induced oxidative stress, mitochondrial damage, and DNA damage can induce a state of senescence-associated stress in some surviving cardiomyocytes, fibroblasts, and endothelial cells (91). These cells may exhibit activation of pathways (such as γ-H2AX, p53/p21^CIP1, p16^INK4A, GATA4, and NF-κB) and initiate early senescence-related secretory programs (92). Unlike mature SASP, which is characterized by typical, sustained low-level secretion during the subacute and chronic phases, these SASP-like signals in the acute phase should be understood as regulatory components within the injury stress network; their secretory profiles highly overlap with those of acute inflammatory factors, although their origins and functions are not entirely identical.

At this stage, SASP-like signals may participate in the amplification and spatial shaping of the acute inflammatory response through multiple mechanisms. First, chemokines such as CCL2 and CXCL1/2 can act in concert with DAMP-induced chemotactic signals to enhance the recruitment of neutrophils and monocytes to the infarct zone and its periphery (93, 94). The recruited immune cells clear necrotic cell debris through phagocytosis, laying the foundation for subsequent tissue repair (95). Recruited monocytes differentiate into macrophages under the influence of the local microenvironment (e.g., high levels of MCP-1, IL-1β, and TNF-α) (96). Second, inflammatory mediators such as IL-1β, TNF-α, and IL-6 can further activate NF-κB and MAPK-related pathways, promoting the maintenance of the pro-inflammatory M1 phenotype in macrophages and amplifying the local inflammatory response (97). M1 macrophages possess potent pro-inflammatory capabilities, which further exacerbate local inflammation and tissue damage (98). Concurrently, exosomes released by M1 macrophages are rich in miR-155. By downregulating the RAC1-PAK1/2 and Sirt1/AMPKα-eNOS signaling pathways, miR-155 inhibits the angiogenic capacity of endothelial cells, exacerbates myocardial infarction damage, and impedes cardiac repair, thereby indirectly amplifying the harmful effects of inflammation (99). Furthermore, OPN expression is upregulated in macrophages, making it a key regulatory factor (100). OPN exerts a bidirectional regulatory effect: in the early phase (3 days), macrophages with high OPN expression primarily participate in leukocyte extravasation, maintaining the inflammatory environment; in the late phase (14 days), OPN expression levels decline, and the fibrotic repair process is initiated (101).

Single-cell sequencing studies have revealed that macrophages exhibit an age-independent senescence-like phenotype following myocardial infarction, characterized by elevated expression of γ-H2AX, IL-1β, and CDKN1A (p21^CIP1), confirming that macrophage SASP is a direct consequence of acute ischemic stress (76). Furthermore, components such as the GATA4–CCN1 axis, TGF-β, MMPs, and extracellular vesicles may participate in matrix degradation, endothelial activation, and the fibroblast response, laying the foundation for subsequent subacute-phase resolution of inflammation, granulation tissue formation, and scar repair (74, 92, 102, 103).

In terms of spatial distribution, during the acute phase, SASP-like signals are more likely to be concentrated in the infarct border zone. The infarct core is characterized primarily by cell necrosis, inflammatory cell infiltration, and tissue clearance; in contrast, the penumbra retains a greater number of sublethally damaged cells. These cells are more prone to DNA damage, mitochondrial dysfunction, and senescence-like changes, and they influence neighboring immune cells, endothelial cells, and fibroblasts via paracrine mechanisms (91). Therefore, the central significance of acute-phase SASP-related regulation lies in its influence on the intensity, extent, and duration of the inflammatory response, as well as the efficiency of its transition to the repair phase, thereby laying the groundwork for the adverse remodeling driven by persistent SASP during the subacute and chronic phases.

#### 3.2.2 Subacute phase of MI

As the acute, DAMP-driven inflammatory burst gradually subsides, the persistent SASP derived from senescent cells begins to play a more prominent role in the subacute phase in the resolution of inflammation, the phenotypic reprogramming of immune cells, ECM remodeling, and scar formation. Studies have shown that senescence-related genes (such as IL-6, IL-1β, and TNF) are significantly upregulated in infarcted myocardium (104). The SASP during this phase acts as a “switch” between inflammation and repair, regulating whether myocardial tissue will undergo excessive fibrosis. On the one hand, by modulating the inflammatory response and the degradation and synthesis of the ECM, it promotes the clearance of necrotic tissue and the orderly formation of scars; on the other hand, if the SASP remains highly expressed, it may lead to chronic inflammation and excessive fibrosis, thereby impairing cardiac function recovery. During the subacute phase following myocardial infarction, the SASP primarily originates from cardiac fibroblasts and some cardiomyocytes that have undergone DNA damage or telomere dysfunction. Both cell types induce a senescence phenotype following ischemia–reperfusion injury, subsequently secreting a series of biologically active factors (57, 59, 105). During the cardiac remodeling and fibrosis phase following myocardial infarction, the SASP is primarily driven by cardiac fibroblasts, with the TGF-β signaling pathway activated, shifting the focus from inflammation to remodeling (88, 106). At this stage, the main components of the SASP include inflammatory cytokines such as IL-1β, IL-6, and TNF-α; growth factors such as TGF-β, PDGFs, and CTGF; and MMPs (26, 107, 108). Among these, IL-1α, as a key upstream initiator of the SASP, acts on senescent cells themselves and their neighboring cells via autocrine or paracrine mechanisms, activating the IL-1 receptor (IL-1R), which in turn triggers the activation of downstream transcription factors such as NF-κB and C/EBPβ. These transcription factors synergistically promote the expression of pro-inflammatory factors such as IL-6 and IL-8, forming a positive feedback loop that further amplifies the SASP effect and induces surrounding cells to enter a senescent state (109, 110). During the subacute phase of myocardial infarction, the anti-inflammatory cytokine IL-10 is persistently upregulated, suppressing excessive inflammatory responses and promoting the transition of tissues to the repair phase (111). TGF-β acts as a central driver of fibrosis by inducing fibroblast activation, promoting collagen deposition, and upregulating TIMPs to inhibit matrix degradation. Concurrently, MMPs degrade the ECM to facilitate tissue remodeling while simultaneously amplifying fibrotic signaling by activating latent TGF-β (112, 113). Concurrently, during the subacute phase, pro-inflammatory signals (IFN-γ and Lipopolysaccharide (LPS)) diminish, while anti-inflammatory/repair-promoting cytokines (IL-4, IL-13, and IL-10) are upregulated. This induces a shift in macrophages from the M1 to the M2a/M2b phenotype, promoting angiogenesis and fibroblast activation, thereby initiating tissue repair (114, 115). Therefore, the SASP in the subacute phase is not merely a harmful signal; rather, it is characterized by persistent, regulatory, and remodeling secretion, participating in the resolution of inflammation, the transition of macrophages to a reparative phenotype, angiogenesis, fibroblast activation, and scar maturation.

#### 3.2.3 Chronic phase of MI

During the chronic phase of MI, senescent cells directly suppress the cytotoxic functions of T cells, NK cells, and macrophages by upregulating immune checkpoint molecules such as PD-L1, HLA-E, and CD47, thereby evading immune attack and persisting in the tissue (116). In the weeks to months following infarction, if senescent cells are not promptly cleared, they will persist in the myocardial microenvironment, establishing a chronic state of SASP secretion. Compared with the subacute phase, the primary clinical significance of chronic-phase SASP lies in its persistence and resistance to resolution. By this stage, the clearance of necrotic tissue, granulation tissue formation, and initial scar repair have largely been completed. Chronic-phase SASP no longer primarily serves to clear inflammation and initiate repair; rather, it becomes a key regulatory factor in maintaining abnormal immune cell infiltration, imbalanced matrix remodeling, paracrine senescence propagation, and the deterioration of ventricular structure. During this period, the composition and function of the SASP undergo significant changes. First, the most notable change is the delay in the resolution of inflammation. In the normal tissue repair process, the inflammatory response should gradually subside after the clearance of necrotic debris; however, persisting senescent cells continuously secrete pro-inflammatory factors such as IL-6, IL-8, and TNF-α, continuously releasing inflammatory signals and establishing a chronic inflammatory state (117). This state of low-grade chronic inflammation impedes cardiac repair and remodeling, directly affecting normal tissue recovery. During the chronic phase of myocardial infarction, senescent fibroblasts secrete IL-10 and TGF-β. IL-10 activates the STAT3 signaling pathway via the IL-10 receptor, while TGF-β induces macrophages to upregulate CD163, downregulate pro-inflammatory factors, and upregulate matrix metalloproteinases and specific chemokines. Together, these mechanisms promote the polarization of macrophages into the M2c subtype, which regulates fibrosis, exerts anti-inflammatory effects, clears apoptotic cells, and modulates matrix remodeling, thereby promoting the stabilization of myocardial scarring (118, 119).

The most destructive feature of chronic-phase SASP lies in its paracrine effects. Damaged cardiomyocytes secrete factors such as IL-6 and TGF-β via the SASP; these factors act on neighboring healthy cells, including fibroblasts, endothelial cells, and cardiomyocytes, inducing them to enter a senescent state (120). This lateral propagation of senescence signals, akin to a vicious cycle, greatly expands the extent of tissue damage. Studies have shown that systemic or non-specific elimination of p16^INK4A-positive cells, thereby inhibiting the release of the SASP, not only significantly improves cardiac function in mice following myocardial infarction but also effectively reduces the area of myocardial scarring (56). This finding directly confirms that the induction of senescence in other cells by senescent cardiomyocytes via the SASP is a central mechanism leading to widespread fibrosis and progressive decline in cardiac function.

The SASP at this stage determines the structural and functional characteristics of scar tissue; if pro-inflammatory SASP factors (such as IL-1β and TNF-α) continue to be overexpressed at this point, it may lead to pathological cardiac remodeling, which in turn triggers heart failure. Therefore, unlike the transient SASP-like response observed during the acute and subacute phases—which may serve a reparative function—the persistent, low-level, pathological SASP observed during the chronic phase is a more suitable target for therapeutic intervention.

### 3.3 Spatial heterogeneity

**Figure 4:**
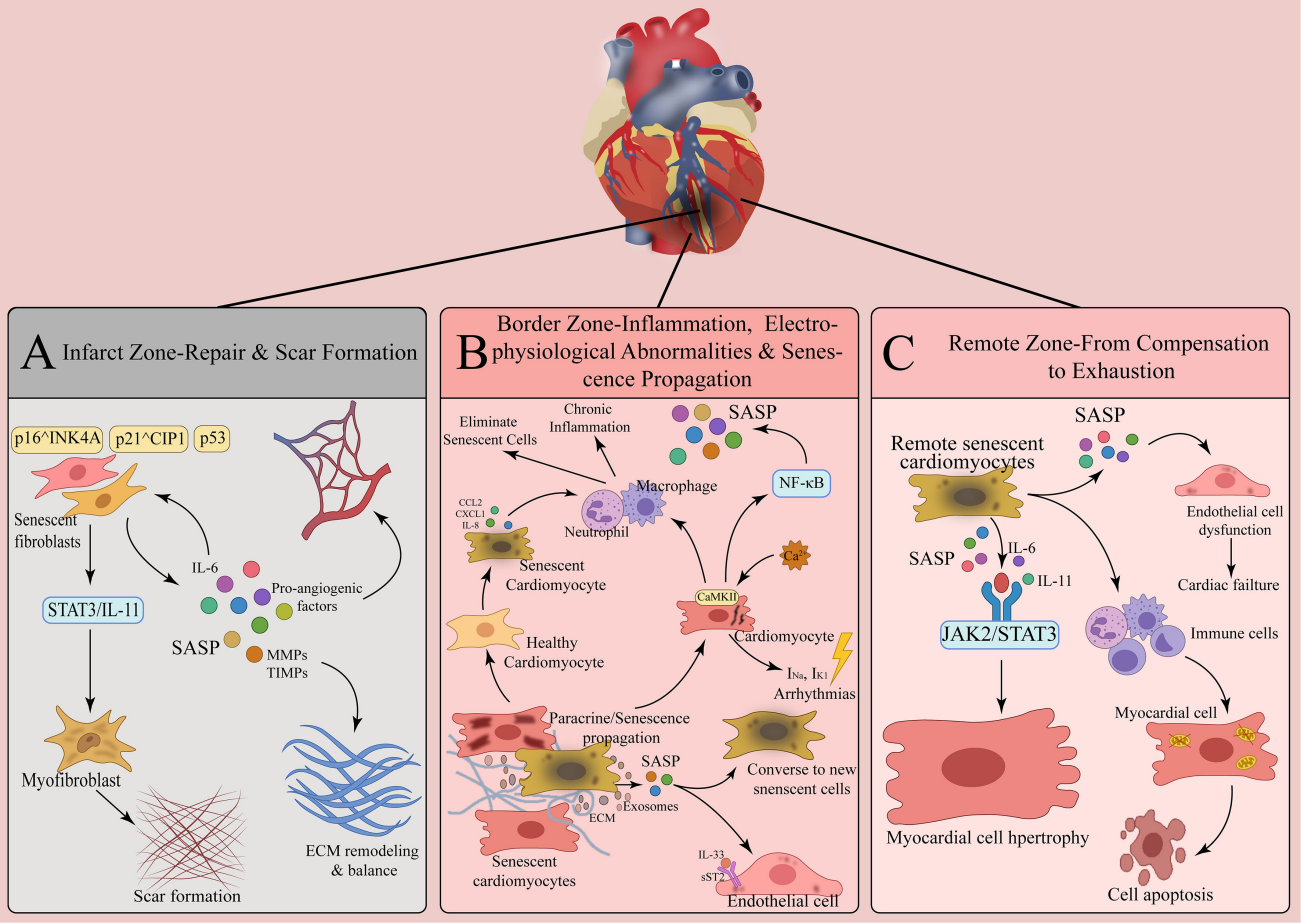
SASP-mediated immune spatial heterogeneity. Following myocardial infarction, the SASP collectively establishes a spatially and temporally ordered, functionally heterogeneous immune landscape through the stabilizing effect of fibrous scar tissue dominated by senescent fibroblasts in the infarct core; the amplification of pathological cascades in the infarct border zone mediated by the bystander effect, inflammation–electrophysiological coupling, and microvascular remodeling; and compensatory remodeling and fibrosis in the distal surviving region triggered by circulating factors. Abbreviations: MI, myocardial infarction; SASP, senescence-associated secretory phenotype; IL, interleukin; MMPs, matrix metalloproteinases; TIMPs, tissue inhibitors of metalloproteinases; NF-κB, nuclear factor-kappa B; CCL2, chemokine ligand 2; CXCL1/2, chemokine ligand 1/2; ECM, extracellular matrix; CaMKII, calcium/calmodulin-dependent protein kinase II; TGF-β, transforming growth factor-beta. **(A)** Infarct Zone; **(B)** The Infarct Margin; **(C)** Distal Non-infarct Area.

From a spatial perspective, post-myocardial infarction cardiac tissue can be divided into the infarct zone, the infarct border zone, and the distal non-infarct zone (121). In these three regions with distinctly different microenvironments, the cellular origins, molecular composition, and biological effects of the SASP exhibit a high degree of functional differentiation, reflecting its context-dependent roles in tissue repair and pathological remodeling (**Figure 4**).

#### 3.3.1 Infarct zone

In the acute phase, the infarct zone undergoes irreversible coagulative necrosis, accompanied by inflammatory cell infiltration and clearance; upon entering the chronic phase, it is replaced by fibrous tissue, forming a scar that leads to ventricular wall thinning and remodeling (122). Among these processes, the core function of the SASP is to maintain the strength of the fibrous scar, replace necrotic myocardium, and counteract high ventricular wall tension.

Senescence markers such as p16^INK4A, p21^CIP1, and p53 are most prominently expressed in the infarct core, where senescent cells accumulate in the highest numbers, collectively contributing to the formation and regulation of the SASP (123). In the infarct core, cardiomyocytes undergo irreversible necrosis due to prolonged ischemia and hypoxia, resulting in massive local cell death and destruction of tissue structure (124). At this stage, fibroblasts become the predominant cell type in the infarct zone and rapidly enter a senescent state, serving as the primary source of the SASP (125).

These senescent fibroblasts secrete large amounts of factors such as TGF-β, IL-1α, and IL-6, which play a dual role (75). Among these, IL-6 is highly expressed in the infarct zone (126). IL-6 promotes the transformation of fibroblasts into myofibroblasts via the STAT3/IL-11 pathway, induces collagen deposition, and forms fibrous scar tissue to replace necrotic myocardial tissue, thereby maintaining the structural integrity of the ventricular wall and preventing ventricular rupture (127, 128). Concurrently, the early SASP environment can also guide the ingrowth of new capillaries by releasing pro-angiogenic factors, delivering essential oxygen and nutrients to the damaged area to support initial granulation tissue formation (129). However, the SASP also participates in regulating the expression of MMPs and their inhibitors (TIMPs), influencing the dynamic balance of the extracellular matrix (130). Furthermore, the persistent presence of the SASP may lead to excessive scar tissue proliferation and stiffness, impairing cardiac diastolic function.

Therefore, in the infarct zone, the core function of the SASP is to drive the reparative fibrotic process, while its immunomodulatory role is relatively indirect, primarily achieving tissue stabilization through the regulation of fibroblast behavior and matrix remodeling.

#### 3.3.2 The infarct margin

As a transition zone, the infarct margin is characterized by marked inflammatory cell infiltration and angiogenesis. Cells on the side adjacent to the infarct undergo irreversible damage and degeneration, while the ischemic penumbra on the outer side remains viable because blood flow has not been completely interrupted, making it a key area for clinical treatment and myocardial repair (131). Although blood perfusion in this region has not been completely interrupted, it remains in a critical state; myocardial cells often undergo sublethal injury, with some entering senescence and beginning to secrete SASP factors (33, 132).

In the infarct border zone, the intensity and duration of the SASP directly determine the nature and consequences of the inflammatory response. Moderate SASP helps clear dead and senescent cells and promotes tissue repair; however, excessive SASP forms a positive feedback inflammatory loop, leading to excessive activation of immune cells and the release of large amounts of inflammatory cytokines such as IL-1β and TNF-α, which further damage the remaining functional myocardial cells and expand the infarct size (133, 134). For example, macrophages, neutrophils, and lymphocytes are recruited to the site by chemokines in the SASP, such as CCL2, CXCL1, and IL-8. While these cells can clear senescent cells and promote tissue repair, they also contribute to the development of chronic inflammation (135). Following myocardial infarction, TGF-β is primarily expressed in the infarct border zone (136). Senescent cells secrete TGF-β, which activates fibroblasts, promoting their differentiation into myofibroblasts that secrete large amounts of collagen (types I and III), leading to compensatory fibrosis (137, 138). Notably, this SASP microenvironment, characterized by the intertwining of intense inflammation and fibrosis, profoundly alters the electrophysiological properties of cells in the infarct border zone.

In the infarct margin of myocardial infarction, calcium/calmodulin-dependent protein kinase II (CaMKII) is activated via a dual mechanism involving Ca^2+^/calmodulin-dependent autophosphorylation and oxidative modification (139). On the one hand, CaMKII upregulates the expression and release of SASP factors via the classical NF-κB transcriptional pathway, forming a positive feedback loop of inflammation; on the other hand, CaMKII alters the inactivation and recovery kinetics of sodium channels (I_Na_), leading to slowed conduction velocity and prolonged effective refractory period, thereby exacerbating local electrical instability (140, 141). This SASP-driven “inflammation–electrophysiological coupling” mechanism makes the infarct margin a key site of origin for post-MI arrhythmias. Concurrently, repolarization heterogeneity in the infarct margin stems from intrinsic electrophysiological remodeling of cardiomyocytes, including downregulation of I_K1_ channel expression, abnormalities in calcium regulation, and the resulting delayed depolarization and prolonged action potential duration, which increase the risk of arrhythmias (142).

Furthermore, the SASP is involved in the remodeling of the ECM and the microvascular microenvironment. In the myocardial infarction border zone, senescent cells transmit senescence signals through various paracrine mechanisms, such as secreting SASP components (e.g., IL-6, IL-1β, and MMP-3), releasing exosomes carrying senescence-associated molecules, and influencing the microenvironment by remodeling the ECM. These signals induce DNA damage, oxidative stress, and inflammatory responses in neighboring cells, thereby promoting secondary senescence in surrounding cells and exacerbating myocardial remodeling and dysfunction (143, 144). Concurrently, the abundance of inflammatory factors in the SASP (such as IL-6 and TNF-α) induces the expression of endothelial adhesion molecules (such as ICAM-1 and VCAM-1), leading to inflammatory cell infiltration and endothelial damage. Among these, soluble ST2 (sST2) can further exacerbate endothelial dysfunction by blocking the cardioprotective effects of IL-33 (145–147).

Therefore, the border zone of the infarct can be regarded as a key node for SASP-mediated immune regulation, and the balance of its inflammatory response determines the final extent of myocardial infarction and the degree of cardiac function recovery.

#### 3.3.3 Distal non-infarct area

The distal non-infarct zone refers to the cardiac region distant from the infarct focus and supplied by non-infarct-related arteries (148). The distal non-infarct zone maintains normal structure during the acute phase; however, upon entering the chronic phase, it undergoes compensatory hypertrophy or fibrosis due to changes in the overall cardiac workload (149). SASP factors not only act on neighboring cells through local diffusion but can also influence distal myocardial cells.

SASP factors secreted by senescent cardiomyocytes in the distal non-infarcted region act synergistically to induce chronic inflammation and fibrosis in this area. Among these, SASP factors such as IL-6 and IL-11 can activate the JAK2/STAT3 pathway, promoting protein synthesis and cell hypertrophy in cardiomyocytes, thereby inducing hypertrophic remodeling in distal cardiomyocytes (150). Endothelial cells in the non-infarcted region undergo functional impairment under the influence of the SASP, further exacerbating cardiac ischemia and hypoxia and promoting the onset of heart failure (151). Additionally, the SASP secreted by senescent cardiomyocytes activates distal immune cells, maintaining a low-grade systemic inflammatory state, which further promotes cardiac interstitial fibrosis and ventricular remodeling (26). In the early post-infarction period, this compensatory hypertrophy helps maintain cardiac output (63). However, in the long term, persistent SASP signaling leads to adverse remodeling, ultimately progressing to heart failure. Concurrently, under sustained inflammation and biomechanical stress, myocardial cells in the distal regions may undergo mitochondrial dysfunction and energy metabolism remodeling, eventually leading to “exhaustion”, manifested as increased apoptosis, impaired autophagy, and decompensated ventricular enlargement (152, 153).

Consequently, under the long-term influence of the SASP, myocardial cells in the distal non-infarcted regions may also undergo exhaustion, leading to ventricular dilation and thinning of the ventricular walls.

## 4 SASP-regulated immune cells and their molecular mechanisms after MI

Inflammation and reparative remodeling following myocardial infarction are driven by a complex network of factors, including innate immunity and cell death mechanisms associated with ischemia–reperfusion injury (154). Therefore, inflammation, repair, and remodeling following myocardial infarction do not rely solely on the SASP and cannot be regarded as mere downstream effects of the SASP.

Within this complex network, the SASP is better defined as a “regulatory layer derived from senescent cells”. Its role is primarily manifested in three aspects. First, the SASP can amplify or prolong existing inflammatory responses, for example, by maintaining immune cell recruitment and pro-inflammatory signaling through factors such as IL-6, IL-1β, TNF-α, and CCL2. Second, the SASP can influence fibroblast activation, ECM turnover, and scar maturation through molecules such as TGF-β, CTGF, MMPs, and TIMPs. Third, the SASP induces secondary senescence in neighboring cardiomyocytes, endothelial cells, and fibroblasts via paracrine mechanisms, thereby transforming local injury into persistent remodeling signals.

**Figure 5:**
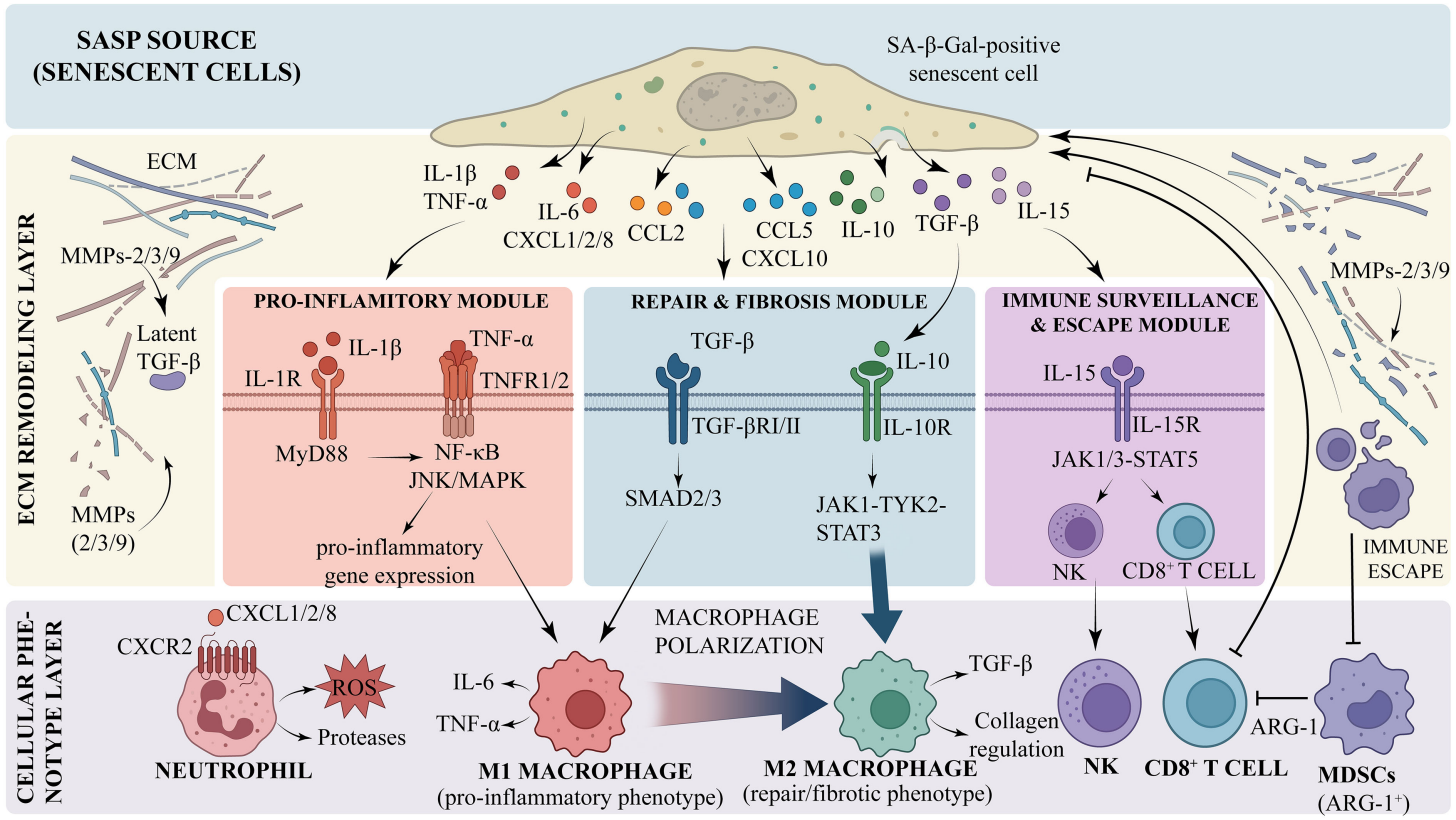
SASP factors produced by senescent cells (such as IL-1β, TNF-α, IL-6, CCL2, and TGF-β) shape the post-infarction immune microenvironment by activating signaling pathways such as NF-κB, JAK–STAT, and MAPK. SASP promotes the recruitment and polarization of neutrophils and M1 macrophages but may also induce M2c macrophages to participate in matrix remodeling. Furthermore, SASP modulates the surveillance functions of NK cells and CD8+ T cells via IL-15, while the accumulation of MDSCs may suppress immune clearance, allowing senescent cells to evade regulation and persist. Abbreviations: MI, myocardial infarction; SASP, senescence-associated secretory phenotype; NF-κB, nuclear factor-kappa B; IL, interleukin; CXCL1/2/8, chemokine ligand 1/2/8; CXCR2, C-X-C motif chemokine receptor 2; MDSCs, myeloid-derived suppressor cells; ARG-1, arginase-1; NK, natural killer; MMPs, matrix metalloproteinases; JAK–STAT, Janus kinase-signal transducer and activator of transcription; TGF-β, transforming growth factor-beta; TNF-α, tumor necrosis factor-alpha.

Based on this, the SASP secreted by senescent cells, through the combined action of multiple cytokines, influences the recruitment, polarization, and functional state of immune cells following myocardial infarction, thereby participating in the regulation of the transition from reparative inflammation to chronic remodeling (**Figure 5**).

In the early stages of myocardial infarction, SASP-associated chemokines can act in concert with DAMPs, ischemia–reperfusion injury, and endothelial cell activation signals to further amplify and prolong the recruitment of innate immune cells. Neutrophils are the first immune cells to be recruited. CXCL1, CXCL2, and CXCL8, acting through the CXCR2 receptor, promote the massive influx of neutrophils into the infarct area, where they clear away necrotic cell debris and create conditions conducive to subsequent repair (155, 156). However, excessive or prolonged neutrophil infiltration leads to the release of reactive oxygen species and proteases, exacerbating myocardial injury (157). Next are monocytes, which are primarily recruited to the heart via CCL2 and CX3CL1 and differentiate into macrophages (158). Macrophages constitute the largest and functionally most complex population of immune cells following myocardial infarction, and their functional state is directly regulated by SASP factors. Factors such as IL-1β and TNF-α induce macrophages to adopt an M1 pro-inflammatory phenotype, causing them to release large amounts of pro-inflammatory cytokines such as IL-1β, IL-6, and TNF-α, thereby further amplifying the local inflammatory response. Conversely, certain anti-inflammatory or repair-related SASP factors may participate in the resolution of inflammation and tissue repair processes, while simultaneously inducing macrophages to switch to the reparative M2 phenotype, thereby supporting tissue repair and anti-inflammatory processes (87, 159, 160).

From the perspective of downstream signaling pathways, IL-1β/IL-1α primarily activates the MyD88–IRAK–TRAF6 axis via IL-1R1, which in turn initiates NF-κB and MAPK signaling (161). This pathway enhances the inflammatory response of neutrophils and monocytes/macrophages, promoting the expression of inflammatory factors such as IL-6, TNF-α, CCL2, and CXCL1/2, thereby amplifying leukocyte recruitment and the pro-inflammatory microenvironment in the infarct zone (162). TNF-α primarily exerts its effects through TNFR1 and TNFR2. TNFR1 tends to activate NF-κB, JNK/p38 MAPK, and cell death-related signaling, promoting the release of pro-inflammatory mediators, ROS, and proteases by macrophages and neutrophils. TNFR2, however, participates in tissue repair and immune regulation in certain immune cells (163). If TNF-α continues to be released from senescent cells after myocardial infarction, it may maintain the pro-inflammatory phenotype of macrophages, enhance the expression of endothelial adhesion molecules, and promote persistent leukocyte infiltration, thereby exacerbating inflammation in the border zone and chronic remodeling. IL-6 can activate the JAK1/2-STAT3 pathway via classical IL-6R/gp130 signaling or trans-signaling, directly affecting the function of macrophages, T cells, and myeloid cells (164). Therefore, during the chronic remodeling phase following myocardial infarction, persistent IL-6 signaling may contribute to the delayed resolution of the inflammatory response and the remodeling of the immune microenvironment; among these, IL-6 derived from senescent cells via the SASP may be one of the key sources maintaining local low-grade inflammation and adverse remodeling (127). Conversely, IL-10 primarily activates the JAK1–TYK2–STAT3 pathway via the IL-10R, inhibiting NF-κB-dependent pro-inflammatory cytokine expression in macrophages and enhancing their ability to phagocytose apoptotic cells and resolve inflammation (165). During the repair phase of myocardial infarction, the IL-10–STAT3 axis helps induce a repair-associated macrophage phenotype, promoting the resolution of inflammation and scar stabilization following the clearance of necrotic tissue; however, when IL-10 is persistently co-expressed with factors such as TGF-β and MMPs, it may also drive M2c-like macrophages to participate in chronic matrix remodeling and fibrosis (119).

TGF-β is a key factor linking immune regulation and fibrotic remodeling. TGF-β activates SMAD2/3 via TGF-βR I/II and can also act on macrophages and T cells through non-classical pathways such as MAPK and PI3K-Akt (113). At the level of immune cell regulation, TGF-β can act in concert with IL-10 to promote the conversion of macrophages to a repair-related phenotype (166). In T cells, TGF-β promotes Treg differentiation and suppresses excessive inflammatory responses (113). However, during the chronic phase, the continuous release of TGF-β by senescent cells can transform the reparative immune response into a pro-fibrotic response, promoting collagen deposition and scar hardening (91, 125). Therefore, the role of TGF-β following myocardial infarction is distinctly phase-dependent. If senescent cells are not cleared in a timely manner, persistent SASP can lead to macrophage dysfunction, with the pro-inflammatory phenotype becoming dominant, thereby inhibiting the clearance of apoptotic cells and exacerbating the vicious cycle of inflammation.

White blood cells play a crucial role in immune surveillance following myocardial infarction. CCL5 and CXCL10 recruit T cells, NK cells, and inflammatory monocytes via CCR5 and CXCR3, respectively; meanwhile, IL-15 enhances the survival, proliferation, and cytotoxicity of NK cells and CD8^+^ T cells through the IL-15R/JAK1/3-STAT5 pathway, enabling them to recognize and eliminate senescent cells (167, 168). However, the accumulation of myeloid-derived suppressor cells (MDSCs) and their inhibitory effects on NK cells weaken this immune clearance capacity. The expansion of MDSCs is primarily driven by SASP factors (such as IL-6, TNF-α, and IL-33), and their functional enhancement depends on the expression of Arg-1; these accumulated MDSCs not only suppress the cytotoxic functions of NK cells and CD8^+^ T cells, creating an immunosuppressive microenvironment, but may also hinder the clearance of senescent cells, thereby promoting chronic inflammation and tissue fibrosis (29, 169). The role of T cells during the recovery phase following myocardial infarction should not be overlooked. CCL5 and CXCL10 in the SASP can recruit CD4+ helper T cells and CD8+ cytotoxic T cells into the heart, while regulatory T cells may also be recruited to suppress excessive immune responses (170, 171). In T cells, IL-6 promotes inflammatory T-cell responses and, under specific conditions, suppresses immune tolerance (88, 172). The SASP can also participate in the regulation of myocardial fibrosis and ventricular remodeling by influencing antigen presentation and altering the activation state of T cells. Dendritic cells, serving as a bridge between innate and adaptive immunity, are similarly influenced by the SASP in their differentiation and activation; factors such as granulocyte–macrophage colony-stimulating factor (GM-CSF) can promote the differentiation of dendritic cell precursors and enhance antigen-presenting function (173).

MMPs such as MMP-2, MMP-3, and MMP-9 can degrade the ECM to form chemotactic matrix fragments that promote the migration of immune cells; they can also release or activate latent TGF-β, thereby altering the repair/fibrosis pathways of macrophages and fibroblasts; simultaneously, they can process chemokines, modifying their stability and activity, and thus influence the spatial distribution of inflammatory cells in the infarct and border zones (174). Therefore, MMPs derived from the SASP are not merely involved in matrix degradation but also participate in post-myocardial infarction immune and structural remodeling through a series of processes.

## 5 Therapeutic strategies targeting SASP and senescent cells

Following myocardial infarction, cardiac tissue suffers from ischemia–reperfusion injury, which not only leads to massive myocardial cell necrosis but also induces surviving cells to enter a persistent state of cellular senescence and secrete the SASP. The persistent presence of the SASP is a key pathological basis driving adverse post-infarction ventricular remodeling, excessive fibrosis, and ultimately heart failure. Therefore, targeting senescent cells and their SASP has become a highly promising therapeutic strategy in the field of cardiovascular regenerative medicine. Current therapeutic strategies are primarily divided into two categories: first, using senolytics to induce apoptosis and directly eliminate senescent cells, and second, using senomorphics to modulate the phenotype of senescent cells, inhibit the synthesis and secretion of the SASP, and thereby mildly alleviate inflammatory responses. At the same time, when targeting senescent cells or the SASP following a myocardial infarction, it is essential to carefully consider the therapeutic window and pathological stage to avoid premature or excessive intervention that could interfere with necessary tissue repair processes. To facilitate a comparison of the target sites, primary mechanisms, and potential stages of application for different intervention strategies, this paper summarizes the currently common therapeutic strategies targeting senescent cells and the SASP, as follows (**Table 1**).

**Table 1:** Key potential therapeutic strategies targeting senescent cells and SASP in myocardial infarction.

Treatment strategies	Exemplary drugs or methods	Key molecular targets	Main effects in MI	Potential intervention window
Senolytics	Dasatinib + quercetin	SCAPs, BCL-2 family, PAI-1, and HIF-1α	Induce apoptosis in senescent cells, reduce myocardial fibrosis, and improve cardiac function	Chronic phase
	Navitoclax (ABT-263)	BCL-2, BCL-xL, and BCL-W	Reduce myocardial stiffness, alleviate compensatory hypertrophy, and improve post-myocardial infarction survival rates	Subacute phase/chronic phase
	Fisetin	PI3K/Akt, NF-κB, p38 MAPK, and BCL-2/BCL-XL	Inhibit age-related inflammation, slow cardiovascular senescence, and improve cardiac function	Subacute phase/chronic phase
Senomorphics	Rapamycin	mTORC1	Reduce the infarct size, inhibit myocardial cell apoptosis, and improve cardiac function	Subacute phase
	Ruxolitinib	JAK–STAT signaling pathway	Reduce the secretion of pro-inflammatory factors	Subacute phase/chronic phase
Epigenetic regulation	JQ1	BRD4	Inhibit SASP expression	Subacute phase/chronic phase
	H151	cGAS–STING pathway	Block the signaling cascade of SASP	Subacute phase
Immune regulation	CAR-T therapy	uPAR’s chimeric antigen receptors	Selectively target and destroy senescent cells	Chronic phase
	ADC	LAMP1	Selectively remove senescent cells	Subacute phase/chronic phase
Metabolic and lifestyle interventions	Metformin/resveratrol	AMPK and SIRT1	Reduce cellular senescence and inhibit the secretion of SASP	Chronic phase
	Aerobic exercise/calorie restriction	Whole-body metabolic reprogramming	Decrease SASP factor levels and reduce the burden of senescence	Long-term prevention

SASP, senescence-associated secretory phenotype; MI, myocardial infarction; SCAPs, senescent cell anti-apoptotic pathways; uPAR, urokinase-type plasminogen activator receptor; LAMP1, lysosome-associated membrane protein 1.

The core mechanism by which senolytics improve post-myocardial infarction function is the clearance of senescent cells, rather than the direct inhibition of SASP synthesis and secretion. Although senescent cells are in a state of cell cycle arrest, they typically activate various senescent cell anti-apoptotic pathways (SCAPs), such as BCL-2/BCL-xL/BCL-W, PI3K/Akt, p21^CIP1, PAI-1/2, HIF-1α, and tyrosine kinase-related pathways to maintain their long-term survival and resist apoptosis (175). By blocking these pro-survival pathways, senolytics restore the sensitivity of senescent cells to mitochondrial-dependent apoptosis, thereby reducing the number of cells contributing to the SASP. As the senescent cell burden decreases, persistent SASP signaling—including IL-6, IL-1β, TNF-α, CCL2, TGF-β, and MMPs—is reduced. This leads to a subsequent alleviation of chronic inflammation, senescence in neighboring cells, abnormal immune cell infiltration, and fibrotic remodeling, ultimately resulting in improved ventricular remodeling and restored cardiac function. The classic regimen for clearing senescent cells is the “D+Q” regimen, which combines dasatinib with quercetin. This regimen synergistically induces apoptosis in senescent cells by inhibiting tyrosine kinases with dasatinib and the PI3K/Akt pathway with quercetin (176). Dasatinib is a multi-targeted tyrosine kinase inhibitor that primarily weakens the anti-apoptotic capacity of senescent cells by inhibiting pro-survival signaling pathways such as RTK/PI3K/Akt. Quercetin primarily acts on SCAP nodes such as PI3K, the BCL-2 family, PAI-1, HIF-1α, and p21^CIP1 while simultaneously inhibiting Akt phosphorylation and promoting the activation of Bax/Bad-related mitochondrial apoptosis pathways (175). Research evidence indicates that periodic administration of “D+Q” in elderly mouse models significantly reduces the expression of the senescence marker p16^INK4A in the heart, alleviates myocardial fibrosis, and improves left ventricular function (177). Furthermore, in humanized animal models of myocardial infarction, D+Q has been shown to restore the function of senescent cardiac progenitor cells (CPCs) and promote cardiac regeneration and repair (178). Navitoclax (ABT-263) is another potent senolytic agent that works by targeting anti-apoptotic proteins in the BCL-2 family; inhibiting BCL-2, BCL-xL, and BCL-W; and disrupting the survival mechanisms that allow senescent cells to resist apoptosis (179). Additionally, in a senescent myocardial infarction model, Navitoclax reduces the senescence burden in senescent cardiomyocytes and that associated with p16^INK4A/p21^CIP1; decreases components of the cardiomyocyte SASP such as TGF-β2; alleviates myocardial hypertrophy, fibrosis, and left ventricular stiffness; and improves post-infarction survival and left ventricular function (62). However, its potential side effect of causing thrombocytopenia limits its use during the acute phase of myocardial infarction, and careful monitoring is required during administration (179). The selective BCL-2 inhibitor ABT-199 selectively inhibits BCL-2, thereby lifting the suppression of BAX/BAK-mediated mitochondrial outer membrane permeabilization and inducing BCL-2-dependent apoptosis in senescent cells (180). However, to date, there have been no reports of clinical studies or applications involving the direct use of ABT-199 in the treatment of post-myocardial infarction. Compared to the aforementioned drugs, fisetin, a natural flavonoid, exhibits lower toxicity. Fisetin targets multiple signaling pathways—including PI3K/AKT, NF-κB, p38 MAPK, and BCL-2/BCL-xL—and reduces the secretion of p16^INK4A-positive senescent cells and SASP factors (such as TNF-α and IL-6). It has demonstrated the potential to clear senescent cells, delay cardiovascular senescence, and improve cardiac function in both *in vitro* and *in vivo* models, making it a more clinically acceptable candidate (181).

Unlike strategies that directly eliminate cells, senomorphics aim to reduce the persistent stimulation of the myocardial microenvironment by pathological SASP by modulating the secretory phenotype of senescent cells, which is particularly important during the chronic inflammatory phase following myocardial infarction. Rapamycin is a classic mTOR inhibitor. Inhibiting the mTOR pathway blocks the translation of IL-1α, thereby attenuating the SASP amplification effect mediated by the IL-1α–NF-κB axis and reducing the production of various SASP factors at the level of protein synthesis (182). Its potential cardioprotective effects may be associated with the inhibition of sustained SASP secretion, reduced inflammatory amplification, improved mitochondrial homeostasis, and attenuated apoptosis. Studies have shown that in a diabetic rabbit model of myocardial ischemia/reperfusion injury, administration of rapamycin during the early reperfusion phase significantly reduces the area of myocardial infarction, improves cardiac function, and inhibits cardiomyocyte apoptosis, confirming its feasibility as a potential therapeutic strategy for myocardial infarction (183). JAK inhibitors, such as ruxolitinib, directly reduce the secretion of key pro-inflammatory factors like IL-6 and IL-1β by blocking the JAK–STAT signaling pathway, thereby significantly alleviating the inflammatory burden caused by senescent cells (184). Additionally, epigenetic regulation is also an effective approach to suppressing the SASP. BET inhibitors, such as JQ1, inhibit the BRD4 protein to block super-enhancer-driven transcription of SASP genes, specifically suppressing SASP expression without affecting cell cycle arrest (185). In recent years, the cGAS–STING pathway has been identified as one of the core mechanisms responsible for sensing leaked cytoplasmic DNA from senescent cells and driving the onset of the SASP. Targeting this pathway with inhibitors such as H-151 can fundamentally block the SASP signaling cascade (186). Furthermore, the use of low-intensity pulsed ultrasound to selectively regulate the expression of specific factors can promote the clearance of senescent cells mediated by M1-type macrophages (187).

In addition to small-molecule drugs, the precise regulation of the immune system is another important research direction. Senescent cells express specific surface markers, which provide ideal targets for immunotherapy. For example, CAR-T cell therapy—which involves genetically engineering T cells to express chimeric antigen receptors targeting the urokinase-type plasminogen activator receptor (uPAR) capable of recognizing uPAR on the surface of senescent cells—enables the specific elimination of senescent cells (188). Concurrently, targeting lysosome-associated membrane protein 1 (LAMP1), which is specifically expressed on the surface of senescent cells, through an antibody–drug conjugate (ADC) strategy achieves selective clearance of senescent cells, offering a new potential avenue for anti-senescence therapy (189). Furthermore, the use of genetically modified mouse models, such as INK-ATTAC or p16^INK4A-3MR, enables scientists to specifically eliminate senescent cells expressing p16^INK4A *in vivo* at specific time points through drug-induced mechanisms. These studies have repeatedly demonstrated in animal experiments that the elimination of senescent cells can significantly improve cardiac function following myocardial infarction (190). Furthermore, small-molecule interventions targeting core SASP transcription factors such as GATA4, NF-κB, and C/EBPβ, as well as inhibitors of the p38 MAPK–MK2 pathway, have demonstrated potential in preclinical studies to reduce inflammation and fibrosis following myocardial infarction (191–194).

In the process of clinical translation, pharmacological treatment strategies often need to be combined with lifestyle interventions to achieve optimal results. Regarding metabolic interventions, drugs such as metformin and resveratrol mimic the effects of calorie restriction by activating the AMPK or SIRT1 pathways, inhibiting mTOR, and enhancing mitochondrial function, thereby reducing ROS-induced cellular senescence and suppressing SASP secretion (109). Lifestyle modifications, such as long-term adherence to aerobic exercise, have been shown to reduce levels of SASP factors in the systemic circulation, thereby indirectly alleviating the burden of senescence-related inflammation in the heart (195). Calorie restriction (CR) or intermittent fasting (IF) suppresses the accumulation of senescent cells at *the systemic level* through metabolic reprogramming (196).

The pathological process following MI progresses rapidly, and the timing of treatment plays a critical role in the efficacy of targeted therapies. It is worth noting that the SASP exhibits marked time dependence. Therefore, selectively targeting senescent cells and their persistent, pathological SASP within an appropriate time window has emerged as a highly promising therapeutic strategy in the field of cardiovascular regenerative medicine. During the acute phase following myocardial infarction, the immune response primarily serves to clear necrotic cells, initiate inflammatory responses, and trigger repair processes; thus, premature use of potent senolytics to eliminate senescent cells may interfere with acute inflammatory clearance and essential tissue repair processes. In the subacute phase, selective modulation using senomorphics or specific SASP pathway inhibitors to limit excessive inflammation or fibrogenic SASP may represent a more regulatory intervention strategy (197). The chronic phase is more suitable for the use of senolytics to clear persisting pathological senescent cells, or for the use of senomorphics to continuously or intermittently inhibit the secretion of pathological SASP, thereby alleviating chronic inflammation and adverse ventricular remodeling (198). Additionally, future precision intervention strategies based on senolytics and senomorphics may need to account for the distinct SASP characteristics of different myocardial infarction regions.

## 6 Prospects

Mortality rates following myocardial infarction and reperfusion remain high, and there are currently no clinically approved treatments available to block the acute injury response following cardiac ischemia (199, 200). Safely and specifically eliminating harmful senescent cells or modulating the SASP is key to reducing inflammation, improving cardiac remodeling, and promoting regeneration. First, given the heterogeneity of senescent cells and the complexity of the SASP, future basic research must thoroughly elucidate the senescence phenotypes and functional differences of various cell lineages at different stages of myocardial infarction. The application of technologies such as single-cell sequencing and spatial transcriptomics will help map a high-resolution profile of senescent cells in the heart, thereby identifying the senescent subpopulations that truly drive pathological processes and their key effector molecules (201). Building on this, the development of novel biomarkers or molecular imaging probes capable of distinguishing between “physiological” and “pathological” senescent cells is a theoretical prerequisite for achieving precise targeting.

Second, regarding the selection of drug targets, future efforts will shift from eliminating senescent cells to regulating their function or selectively eliminating specific subpopulations. Furthermore, targeted protein degradation strategies based on Proteolysis Targeting Chimera (PROTAC) technology, or gene editing tools based on clustered regularly interspaced short palindromic repeats (CRISPR)-Cas9, also offer new possibilities for precisely intervening in pathological processes and promoting cardiac repair (202, 203). These methods are expected to block the adverse effects of senescent cells on the microenvironment while preserving their beneficial functions.

Regarding drug delivery systems, breakthroughs in cardiac-specific targeting strategies will be key to enhancing efficacy and reducing systemic toxicity. Currently, most targeted drugs are administered systemically, which not only affects senescent cells throughout the body but also increases the risk of immune system damage. Additionally, due to impaired blood flow in the infarct core, drug efficacy is limited. Therefore, the development of delivery systems capable of directly targeting the heart or the infarct area is a current research focus. For example, designing smart, responsive nanomedicines that exploit the unique microenvironment of the myocardial infarction site (such as acidic pH, elevated reactive oxygen species levels, and enhanced matrix metalloproteinase activity) can enable controlled-release delivery to the infarct site (204). In the future, precise stratification based on patients’ pathophysiological characteristics (such as shock risk and microcirculatory function) may enable the individualized selection of strategies—such as mechanical circulatory support or delayed reperfusion—to reduce infarct size and lower the incidence of heart failure (205). Additionally, the use of stem cells for myocardial regeneration and the optimization of mechanical circulatory support devices (such as left ventricular assist devices) may help reduce complications, improve risk stratification for heart transplantation, and enhance the precise application of surgical interventions in heart failure management (206).

## 7 Conclusion

Cardiac repair and remodeling following myocardial infarction constitute a highly complex pathophysiological process, in which cellular senescence and its associated SASP play a critical dual regulatory role. The molecular characteristics and functional alterations observed, as different types of cardiac cells enter a senescent state after myocardial infarction, reveal the phasic evolution of the SASP across the temporal dimension. The SASP evolves from pro-inflammatory responses and necrotic tissue clearance in the acute phase, to tissue repair and fibrosis regulation in the subacute phase, and finally to persistent inflammation and the driving of adverse remodeling in the chronic phase; it also exhibits significant functional differentiation across spatial dimensions among the infarct zone, the infarct border zone, and the distal non-infarct zone. Concurrently, by finely regulating the recruitment, polarization, and functional states of immune cells through multiple signaling pathways, the SASP demonstrates its central role in the spatiotemporal dynamic network of the immune system. Based on these mechanisms, therapeutic strategies targeting senescent cells and their SASP have shown potential in preclinical studies to improve cardiac function, reduce fibrosis, and promote repair. Currently, the safe and specific removal of harmful senescent cells or the modulation of the SASP is key to reducing inflammation, improving cardiac remodeling, and promoting regeneration. At the same time, preventing recurrent myocardial infarction and implementing personalized precision therapy are essential for ensuring long-term stability in patients’ lives.
